# Mitigating heat stress in Garut lambs: Synergistic effects of Lemuru fish oil, vitamin E, and selenium on antioxidant defense, hematology, and physiological responses

**DOI:** 10.14202/vetworld.2025.2230-2240

**Published:** 2025-08-09

**Authors:** Aeni Nurlatifah, Dewi Apri Astuti, Herdis Herdis, Iis Arifiantini, Fitra Aji Pamungkas, Santoso Santoso, Didid Diapari, Pradita Iustitia Sitaresmi, Enny Tantini Setiatin, Athar Manabi Diansyah

**Affiliations:** 1 Department of Animal Nutrition and Feed Science, Faculty of Animal Science, Gadjah Mada University, 55281 Yogyakarta, Indonesia; 2 Department of Nutrition and Feed Technology, Faculty of Animal Science, Bogor Agricultural University, 16680 Bogor, Indonesia; 3 Research Center for Animal Husbandry, National Research and Innovation Agency, 16915 Jakarta, Indonesia; 4 Department of Department of Clinical, Reproductive, and Pathology, Veterinary Medicine and Biomedicine College, Bogor Agricultural University, 16680 Bogor, Indonesia; 5 Department of Animal Production, Faculty of Animal and Agricultural Sciences, Diponegoro University, 50275 Semarang, Indonesia; 6 Department of Animal Production, Faculty of Animal Science, Hasanuddin University, 90245 Makassar, Indonesia

**Keywords:** antioxidants, Garut sheep, heat stress, hematology, lemuru fish oil, omega-3, oxidative stress, physiological response, selenium, Vitamin E

## Abstract

**Background and Aim:**

Tropical climates expose livestock to prolonged heat stress, leading to compromised health, growth, and productivity. Nutritional interventions using omega-3 fatty acids and antioxidants may improve resilience in such environments. This study evaluated the effects of lemuru fish oil (LO), vitamin E, and selenium – individually and in combination – on hematological, physiological, antioxidant, and growth parameters of Garut lambs exposed to tropical heat stress.

**Materials and Methods:**

Forty male Garut lambs (23.52 ± 2.51 kg; 9–10 months old) were randomly assigned to five dietary groups: Control (CNT), LO (6%, LO), LO + 500 IU Vitamin E, LO + 0.5 ppm selenium, and LO + both Vitamin E and selenium (LOES). The 60-day trial took place during the dry season under severe to extreme heat stress conditions (temperature-humidity index: 25.13–40.07). Parameters assessed included nutrient intake, physiological responses, blood hematology, antioxidant status, and growth performance.

**Results:**

Supplementation with LO and antioxidants significantly improved hematological indicators (e.g., hemoglobin), enhanced antioxidant defenses (e.g., increased superoxide dismutase [SOD] and glutathione, reduced malondialdehyde [MDA], and cortisol levels), and stabilized physiological responses (e.g., lower rectal temperature and heart rate). The LOES group showed the most pronounced improvements: SOD increased by 107%, MDA decreased by 62%, and cortisol levels were reduced by 28% compared to the CNTs. However, no significant differences were observed in average daily gain or feed efficiency.

**Conclusion:**

The combination of LO with Vitamin E and selenium effectively mitigated heat-induced oxidative and physiological stress in Garut lambs. Although growth parameters remained unchanged, the improved physiological and antioxidant status suggests that these supplements may be valuable functional feed additives for enhancing animal welfare and resilience under heat stress. Further research is warranted to investigate the long-term effects on productivity and reproduction.

## INTRODUCTION

Tropical environments impose significant chall-enges on livestock productivity, primarily due to the persistent threat of heat stress. These adverse con-ditions are exacerbated by the limited availability of high-quality forage, which is typically characterized by high dry matter (DM) content and elevated crude fiber levels in tropical regions [1]. Such nutritional constr-aints, coupled with intense heat, particularly during the dry season, complicate livestock management, impair animal welfare, and reduce reproductive efficiency and overall performance. During critical physiological stages, such as growth or lactation, heat stress leads to reduced feed intake and increased metabolic heat production, both of which ultimately result in diminished productivity [2]. Elevated core body temperatures interfere with key physiological mechanisms, further reducing performance in livestock [3]. In addition, thermal stress disrupts immune function and other physiological processes, thereby contributing to systemic imbalances and compromised health [4]. Coll-ectively, these effects translate into significant econ-omic losses across livestock production systems. As climate change continues to escalate the intensity and frequency of heat stress events, the sustainability of animal agriculture, especially in tropical zones, is under increasing threat [5, 6]. In response, the development of effective climate-adaptive livestock strategies has become an urgent priority.

Garut sheep, an indigenous breed from Indon-esia, exhibit notable adaptation to tropical climates and are valued for their productivity and resilience [7]. Nevertheless, extreme environmental fluctuations resulting from global climate change can overwhelm their physiological tolerance, leading to stress-related dysfunction and declines in productivity. Among vari-ous environmental challenges, heat stress remains the most critical factor affecting small ruminants in tropical regions [8]. Its adverse impacts include reduced growth rates, reproductive performance, and milk yield, as well as alterations in hematological parameters and biochemical profiles [9]. Understanding the physiol-ogical consequences of heat stress and exploring nutrit-ional countermeasures are vital to formulating effective mitigation strategies.

Dietary interventions using omega-3-rich feed-stuffs and antioxidants have gained attention as pro-mising approaches to alleviate heat stress-induced damage in ruminants [10]. Supplementation with lipid sources rich in omega-3 and omega-6 fatty acids, such as lemuru fish oil (LO), can enhance nutrient digestibility and absorption [11]. These fatty acids play critical roles in supporting membrane fluidity, neural development, and thermoregulation, which are ess-ential for adaptation under elevated environmental temperatures [12]. Furthermore, antioxidants such as Vitamin E and selenium contribute to reducing heat-stress-induced inflammation by neutralizing reac-tive oxygen species (ROS) and preserving gut barrier integrity [13]. Both nutrients are integral to immune function, oxidative metabolism, and energy utilization in ruminants, supporting overall health and resilience under stress [14].

Although individual supplementation with omega-3 fatty acids, Vitamin E, or selenium has shown promise in enhancing oxidative status and physiological resilience in ruminants under thermal stress, most existing studies have focused on their effects in isolation. There is limited research exploring the synergistic or comparative impact of these nutrients, especially in the context of sheep reared under severe tropical heat stress. Moreover, the response of indigenous sheep breeds like Garut sheep, well-adapted to tropical climates but still vulnerable to extreme heat, remains poorly understood in this regard. Current knowledge is insufficient to determine whether the combined use of LO, Vitamin E, and selenium offers additive or redundant benefits. In addition, while antioxidant biomarkers and physiological parameters have been assessed in cattle and goats under heat stress, comprehensive studies evaluating these markers alongside hematology and growth performance in Garut lambs are lacking. This gap limits the development of evidence-based nutritional interventions specifically targeted to tropical sheep production systems.

This study aimed to evaluate the effects of LO, alone and in combination with Vitamin E and selenium, on the physiological responses, hematological profiles, antioxidant status, and growth performance of Garut lambs reared under tropical heat stress conditions. Specifically, it investigated whether combining these functional feed additives would confer superior ben-efits compared to single nutrient supplementation in mitigating oxidative damage and enhancing therm-otolerance. The findings aim to inform practical feeding strategies that reduce the adverse impacts of climate-induced heat stress on small ruminant production syst-ems in the tropics.

## MATERIALS AND METHODS

### Ethical approval

All procedures were reviewed and approved by the Ethical Committee of the National Research and Innovation Agency (BRIN), under approval number 01112023000022.

### Study period and location

The study was conducted during the dry season (August and September 2023) at the Cikarawang Farm, Bogor, West Java, Indonesia (6°32’38.3”S, 106°43’35.6”E). The trial lasted 57 days, including a 7-day adaptation period and a 50-day treatment phase.

### Experimental animals, housing, and diets

A total of 40 male Garut lambs (average body weight: 23.52 ± 2.51 kg; aged 9–10 months) with uniform body condition scores and fleece lengths were selected for the study. Animals were housed individually in 1 m^2^ elevated pens equipped with plastic slatted polypropylene flooring and wooden slatted walls for natural ventilation. Each pen was covered with asbestos roofing and had a height of 2.5 m. No artificial environmental control (CNT) (e.g., fans or sprinklers) was used.

Animals were fed a mixed ration based on a 30:70 forage-to-concentrate ratio (DM basis), adjusted to 3.8% of body weight. Pennisetum purpureum cv. Thailand was used as the forage source. The feeding schedule included morning concentrate feeding at 07:00 a.m. and afternoon forage feeding at 02:00 p.m. Clean drinking water was provided *ad libitum*.

The lambs were randomly divided into five dietary treatment groups (n = 8 per group):

- CNT: CNT concentrate
- LO: CNT + 6% LO
- LO + 500 IU Vitamin E (LOE)
- LO + 0.5 ppm selenium (LOS)
- LOES: LOE + 0.5 ppm selenium.

Tables 1 and 2 provide the detailed compo-sition and nutritional content of the rations. LO was thoroughly mixed with the concentrate. Vitamin E (DL-alpha-tocopheryl acetate, Zhejiang Medicine Co., Ltd., China) and selenium (sodium hydrogen selenite, Merck, Germany) were administered orally every mor-ning. Concentrates were prepared weekly and stored in airti-ght containers to minimize oxidation. Body weight was recorded biweekly to calculate average daily gain (ADG) and feed efficiency (FE).

**Table 1:** Composition of the control concentrate and additional supplements (lemuru oil, Vitamin E, and selenium) for each treatment group (dry matter basis).

Feed ingredient (%)	CNT	LO	LOE	LOS	LOES
Rice bran	31.71	31.71	31.71	31.71	31. 71
Copra meal	13.16	13.16	13.16	13.16	13.16
Pollard	4.32	4.32	4.32	4.32	4.32
Cassava	9.73	9.73	9.73	9.73	9.73
Corn gluten feed	5.18	5.18	5.18	5.18	5.18
Coffee husk	27.05	27.05	27.05	27.05	27.05
Molasses	8.31	8.31	8.31	8.31	8.31
CaCO_3_	0.15	0.15	0.15	0.15	0.15
Salt	0.29	0.29	0.29	0.29	0.29
Lemuru fish oil	-	6	6	6	6
Vitamin E	-	-	500 IU	-	500 IU
Selenium	-	-	-	0.5 ppm	0.5 ppm

CNT=Control concentrate, LO=Control concentrate + lemuru fish oil 6%, LOE=LO + Vitamin E 500 IU, LOS=LO + Se 0.50 ppm, LOES=LO + Vitamin E 500 IU + Se 0.50 ppm

**Table 2:** Chemical composition of the diet concentrates for each treatment group (%, dry matter basis).

Nutrient content (%)	CNT	LO	LOE	LOS	LOES
Crude protein	11.07	10.44	10.44	10.44	10.44
Crude fiber	13.82	13.03	13.03	13.03	13.03
Crude fat	4.02	9.45	9.45	9.45	9.45
Nitrogen free extract	61.15	57.69	57.69	57.69	57.69
Total digestible nutrients	65.06	68.75	68.75	68.75	68.75
Calcium	0.46	0.44	0.44	0.44	0.44
Phosphor	1.17	1.10	1.10	1.10	1.10

CNT=Control concentrate, LO=Control concentrate + lemuru fish oil 6%, LOE=LO + Vitamin E 500 IU, LOS=LO + Se 0.50 ppm, LOES=LO + Vitamin E 500 IU + Se 0.50 ppm

### Temperature-humidity index (THI) monitoring

Ambient temperature and relative humidity (RH) were measured 3 times daily (07:00, 12:00, and 16:00) using a digital thermo-hygrometer 30.5002 (TFA Dostmann GmbH & Co.KG, Wertheim, Germany) susp-ended at the lambs’ shoulder height. The THI was calculated using the formula by Marai *et al*. [16]: THI = T – ([0.31–0.31 × RH] × (T–14.4]), where T is ambient temperature (°C) and RH is relative humidity (%). Heat stress categories were defined as follows:

- THI ≤22.2: No heat stress
- THI 22.2–23.3: Moderate
- THI 23.3–25.6: Severe
- THI >25.6: Extreme heat stress

### Feed intake and nutrient analysis

Daily feed intake and refusals were recorded to calculate DM intake (DMI). Nutrient intake (e.g., CP, CF, and nitrogen-free extract [NFE]) was computed by multiplying DMI with the respective nutrient conce-ntrations obtained from proximate analysis. NFE was calculated by difference. Total digestible nutrients (TDN) were estimated following Jayanegara *et al*. [17].

### Physiological measurements

From days 53 to 60, physiological parameters, including rectal temperature, heart rate, and respiratory rate, were recorded 3 times daily (07:00, 12:00, and 04:00).

- Respiratory rate was assessed by counting flank movements using a stopwatch.
- Heart rate was measured using a prestige medical stethoscope.
- Rectal temperature was taken with a Terumo digital thermometer.

To minimize handling stress, measurements were taken by the same trained observer on manually restr-ained, standing lambs.

### Blood sampling and laboratory analyses

On day 60, blood samples were collected from the jugular vein at 08:00 a.m., post-feeding.

- 1 mL was collected inethylenediaminetetraacetic acid tubes (Vaculab EDTA K3, OneMed, Surabaya, Indonesia) for hematological analysis
- 3 mL was collected in plain vacutainers for serum separation. Serum was obtained by centrifugation at 9,046 × *g* for 15 min at 4°C and stored at −20°C.
- The following assays were performed:
- Oxidative stress and antioxidant markers: Malon-dialdehyde (MDA), Superoxide dismutase (SOD) (BioVision, USA); glutathione peroxidase, catalase (CAT), and vitamin E (Cusabio Biotech Co., Ltd., China).
- Hematological indices: Packed cell volume (PCV), hemoglobin (Hb), erythrocyte, and leukocyte cou-nts analyzed according to Buttarello [18].

### Growth performance evaluation

Lambs were weighed at the start of the experiment and subsequently at weekly intervals.

- ADG was calculated as total weight gain divided by the number of days
- FE was expressed as ADG per unit of DMI.

### Statistical analysis

A completely randomized design was used with eight replicates per treatment. Initial body weight ser-ved as a covariate (blocking factor).

- Data normality and homogeneity were checked before analysis
- One-way analysis of variance was used for most parameters using Statistical Analysis System (SAS) (v9.4, SAS Institute Inc., USA)
- Physiological data were analyzed using a two-way factorial design with dietary treatment and time of day as factors
- Results are presented as means ± standard deviation
- Duncan’s multiple range test was applied for *post hoc* comparisons when significant differences were found (p < 0.05).

No animals were excluded or lost during the study.

## RESULTS

### Effects on nutrient intake

The effects of LO, selenium, and Vitamin E on nutrient intake in male lambs are summarized in Table 3. Significant treatment effects were observed (p < 0.01). DMI was reduced by approximately 13% in lambs receiving LO and its combinations with Vitamin E (LOE), selenium (LOS), or both (LOES) compared to the CNT. Similarly, intake of crude protein, crude fiber, and NFE significantly decreased (p < 0.01) across all supplemented groups. In contrast, crude fat intake was significantly higher (p < 0.01) in all groups receiving LO and its antioxidant combinations compared to the CNT. TDNs were reduced in the LOE group (p < 0.05), while LO, LOS, and LOES groups exhibited similar TDN values.

**Table 3:** Effects of lemuru fish oil, selenium (Se), and Vitamin E on nutrient consumption in male lambs.

Nutrient consumption (g/h/day)	CNT	LO	LOE	LOS	LOES
Dry matter	943.45 ± 50.50^a^	780.61 ± 68.07^b^	790.0 ± 44.10^b^	809.38 ± 44.40^b^	814.87 ± 47.27^b^
Crude protein	91.58 ± 5.12^a^	79.99 ± 10.38^b^	75.42 ± 5.22^b^	80.10 ± 8.16^b^	78.56 ± 7.22^b^
Crude fiber	114.34 ± 6.39^a^	99.92 ± 12.97^b^	94.19 ± 6.65^b^	100.05 ± 10.19^b^	98.13 ± 9.02^b^
Crude fat	33.25 ± 1.85^b^	75.09 ± 9.75^a^	70.79 ± 4.90^a^	75.19 ± 7.66^a^	73.74 ± 6.78^a^
Nitrogen free extract	506.01 ± 28.31^a^	442.14 ± 57.40^b^	416.80 ± 28.88^b^	442.72 ± 45.09^b^	434.20 ± 39.93^b^
TDN	538.28 ± 30.12^a^	514.01 ± 66.74^ab^	484.56 ± 33.58^b^	514.70 ± 52.43^ab^	504.79 ± 46.42^ab^

CNT=Control concentrate, LO=Control concentrate + lemuru fish oil 6%, LOE=LO + Vitamin E 500 IU, LOS=LO + Se 0.50 ppm, LOES=LO + Vitamin E 500 IU + Se 0.50 ppm. The same superscript in the same row indicates significant differences (p < 0.01) for treatments. TDN=Total digestible nutrients

### Effects on physiological parameters

As shown in Table 4, the ambient temperature, RH, and THI indicated extreme heat stress during the noon and afternoon periods. Consequently, Table 5 presents the effects of dietary treatments on physiological responses under these conditions. Both heart rate and rectal temperature were significantly affected by treatment (p < 0.01), while respiratory rate was not (p > 0.05). Compared to the CNT group (120.35 beats/min), heart rate was significantly reduced in the LO group (103.39 beats/min) and further decreased in the LOES group (96.17 beats/min). However, supplementation with either Vitamin E (LOE) or selenium (LOS) alone did not produce further reductions beyond that of LO.

**Table 4:** The ambient temperature (Ta), RH, and THI at the farm during the study period.

Microclimate	Period		

	Morning	Noon	Afternoon
Mean temperature (°C)	25.12	40.07	32.42
Maximum temperature (°C)	24.20	39.01	26.1
Minimum temperature (°C)	26.6	42.2	35.8
RH %	94.00	37.14	61.57
THI^1^	25.12	40.07	32,42
THI categories	Severe heat stress	Extreme severe heat stress	Extreme severe heat stress

1 Formulation and categories according to Marai *et al*. [16]. RH=Relative humidity, THI=Temperature-humidity index

**Table 5:** Effects of lemuru fish oil, selenium (Se), and vitamin E on the physiological response of male lambs.

Parameters	Time observation	CNT	LO	LOE	LOS	LOES	Average
Heart rate (beats/min)	Morning	116.45 ± 7.06	93.55 ± 11.90	91.39 ± 8.25	89.59 ± 18.22	88.05 ± 11.93	99.20^B^
	Noon	125.53 ± 11.43	107.06 ± 8.03	102.6250 ± 9.37	100.81 ± 12.24	97.78 ± 10.56	110.01^A^
	Afternoon	118.86 ± 10.34	109.57 ± 9.22	105.7488 ± 9.42	105.63 ± 14.03	102.68 ± 13.74	109.74^A^
	Average	120.35 ± 10.31^A^	103.39 ± 11.81^B^	99.92 ± 10.68^BC^	98.57 ± 16.00^BC^	96.17 ± 13.17^C^	
Respiratory rate (breaths/min)	Morning	40.42 ± 1.68	38.50 ± 2.76	37.25 ± 3.76	39.00 ± 3.65	37.41 ± 3.59	38.86^C^
	Noon	77.58 ± 3.29	81.00 ± 8.35	78.38 ± 9.34	75.74 ± 9.43	69.69 ± 13.85	76.32^A^
	Afternoon	52.29 ± 4.26	52.01 ± 1.81	57.42 ± 3.80	54.82 ± 7.53	55.80 ± 3.89	54.29^B^
	Average	56.76 ± 15.9	55.00 ± 18.23	57.68 ± 18.15	54.50 ± 16.18	54.30 ± 15.79	
Rectal temperature (°C)	Morning	37.62 ± 0.43	37.41 ± 0.37	37.35 ± 0.61	37.10 ± 0.28	37.25 ± 0.47	37.40^B^
	Noon	38.77 ± 0.38	38.41 ± 0.14	38.41 ± 0.23	38.45 ± 0.21	38.55 ± 0.11	38.57^A^
	Afternoon	38.93 ± 0.52	38.48 ± 0.13	38.42 ± 0.24	38.33 ± 0.28	38.40 ± 0.22	38.58^A^
	Average	38.43 ± 0.72^A^	38.10 ± 0.55^B^	38.06 ± 0.64^B^	38.03 ± 0.64^B^	38.05 ± 0.67^B^	

CNT=Control concentrate, LO=Control concentrate + lemuru fish oil 6%, LOE=LO + Vitamin E 500 IU, LOS=LO + Se 0.50 ppm, LOES=LO + Vitamin E 500 IU + Se 0.50 ppm. The same superscript in the same row indicates significant differences (p < 0.01) for treatments. The same superscript in the same column indicates significant differences (p < 0.01) for observation time

Rectal temperature was also significantly lower (p < 0.01) in all supplemented groups (LO, LOE, LOS, and LOES) compared to the CNT (38.43°C), ranging between 38.03°C and 38.10°C. Across all groups, heart rate and rectal temperature were highest at noon and afternoon (p < 0.01), indicating diurnal variation. Respiratory rate was significantly elevated at noon, approximately double the morning values, before declining to intermediate levels by the afternoon.

### Effects on hematological profiles

The hematological outcomes are shown in Table 6 [1–6]. Erythrocyte counts tended to increase (p = 0.057) with LO supplementation but declined when combined with antioxidants, approaching CNT levels. Hb levels were significantly increased (p < 0.01) in all supplemented groups compared to CNT (8.62, 9.12, 8.55, and 8.48 vs. 7.79 g/dL, respectively).

**Table 6:** Effects of lemuru fish oil, selenium (Se), and Vitamin E on blood hematology in male lambs.

Parameters	CNT	LO	LOE	LOS	LOES	References value
PCV (%)	24.93 ± 3.32	25.14 ± 2.95	27.00 ± 2.65	24.00 ± 1.44	24.66 ± 1.84	24–41 [1]
Erythrocyte (10^6^/mm^3^)	10.08 ± 2.80	13.18 ± 2.31	11.19 ± 1.68	11.86 ± 2.80	11.83 ± 3.20	9–15 [2]
Hemoglobin (g/dL)	7.79 ± 0.79^b^	8.62 ± 0.66^a^	9.12 ± 0.84^a^	8.55 ± 0.29^a^	8.48 ± 0.37^a^	8.5–12 [3]
MCV (fl)	25.76 ± 6.49^a^	19.43 ± 3.07^b^	21.76 ± 3.03^b^	21.12 ± 4.14^b^	21.87 ± 4.42^b^	25–35 [2]
MCH (pg)	8.18 ± 1.96^a^	6.62 ± 0.94^b^	7.90 ± 1.03 ^a^	7.49 ± 1.36 ^ab^	7.57 ± 1.77 ^ab^	8–12 [4]
MCHC (g/dL)	32.29 ± 6.17^b^	34.50 ± 2.50^ab^	34.46 ± 1.75^ab^	35.73 ± 1.78^a^	34.50 ± 1.59^ab^	31–34 [2]
Leucocyte (10^3^/mm^3^)	9.02 ± 1.91	10.68 ± 0.97	11.06 ± 1.86	10.95 ± 1.85	10.30 ± 1.64	4–12 [2]
Limfosit (L) (%)	47.77 ± 12.88^b^	50.72 ± 3.55^ab^	59.02 ± 8.59^a^	50.25 ± 7.76^ab^	50.37 ± 10.09^ab^	43–65 [5]
Monosit (%)	1.84 ± 0.68^a^	3.31 ± 1.34^b^	1.25 ± 0.54^a^	1.86 ± 0.78^a^	1.46 ± 0.92^a^	0.5–6 [6]
Neutrofil (N) (%)	40.01 ± 12.28	39.92 ± 4.05	34.91 ± 8.69	36.95 ± 10.81	41.12 ± 10.43	26–42 [5]
Eosinofil (%)	6.69 ± 2.57	5.23 ± 1.79	4.06 ± 1.41	3.74 ± 0.64	5.82 ± 2.11	2–10 [5]
Basophil (%)	0.97 ± 0.21	0.85 ± 0.06	0.74 ± 0.32	0.96 ± 0.12	0.75 ± 0.26	0–3 [5]
N/L (%)	1.43 ± 0.49^a^	0.79 ± 0.11^b^	0.59 ± 0.26^b^	0.74 ± 0.31^b^	0.87 ± 0.36^b^	

CNT=Control concentrate, LO=Control concentrate + lemuru fish oil 6%, LOE=LO + Vitamin E 500 IU, LOS=LO + Se 0.50 ppm, LOES=LO + Vitamin E 500 IU + Se 0.50 ppm. The same superscript in the same row indicates significant differences (p < 0.05) for treatments. PCV=Packed cell volume, MCV=Mean corpuscular volume, MCH=Mean corpuscular hemoglobin, MCHC=Mean corpuscular hemoglobin concentration

Mean corpuscular volume (MCV) was significantly reduced (p < 0.05) in LO, LOE, LOS, and LOES groups relative to CNT (19.43–21.87 vs. 25.76 fL). Mean corpuscular Hb (MCH) values were significantly higher in CNT and LOE than in LO, while LOS and LOES fell within a similar intermediate range. MCH concentration (MCHC) was significantly increased (p < 0.01) in the LOS group compared to CNT (35.73 vs. 32.29 g/dL). There were no significant differences (p > 0.05) in PCV or leukocyte counts among groups.

### Effects on leukocyte differentiation

Dietary treatments significantly influenced leuk-ocyte profiles (Table 6) [1–6]. Lymphocyte percentages were highest (p < 0.05) in the LOE group (59.02%) compared to CNT (47.77%). Monocyte percentages were significantly higher (p < 0.01) in the LO group compared to the other treatments. No significant differences were observed in neutrophil, eosinophil, or basophil counts across groups (p > 0.05). The neutrophil-to-lymphocyte (N/L) ratio was significantly reduced (p < 0.05) in all supplemented groups (LO, LOE, LOS, and LOES) compared to CNT (0.79, 0.59, 0.74, and 0.87 vs. 1.43), indicating reduced physiological stress.

### Effects on antioxidant status

Table 7 shows the antioxidant responses to dietary treatments. MDA levels were significantly reduced (p < 0.05) in all supplemented groups relative to CNT (1.74–2.77 vs. 5.13 nmol/μL), indicating lower lipid peroxidation. SOD activity was significantly increased (p < 0.05), with the highest value observed in the LOES group (63.06 U/mL), more than double that of CNT (30.51 U/mL). CAT activity was not significantly affected (p > 0.05).

**Table 7:** Effects of lemuru fish oil, selenium (Se), and vitamin E on the antioxidant status of male lambs.

Antioxidant status	CNT	LO	LOE	LOS	LOES
Malondialdehyde (nmol/µL)	5.13 ± 0.47^b^	1.74 ± 0.89^a^	2.77 ± 1.09^a^	2.55 ± 0.64^a^	1.97 ± 0.71^a^
Superoxide dismutase (U/mL)	30.51 ± 2.77^c^	57.67 ± 13.36^ab^	43.11 ± 15.07^b^	52.96 ± 9.22^ab^	63.06 ± 13.79^a^
Catalase (ng/mL)	0.16 ± 0.01	0.15 ± 0.03	0.14 ± 0.02	0.12 ± 0.01	0.13 ± 0.02
Glutathione (ng/mL)	5.72 ± 0.69^b^	6.61 ± 4.16^b^	20.87 ± 5.11^a^	24.57 ± 4.96^a^	25.52 ± 5.66^a^
Cortisol (ng/mL)	71.69 ± 3.81^c^	62.80 ± 6.56^b^	63.11 ± 4.02^b^	60.22 ± 7.87^b^	51.31 ± 11.60^a^
Vitamin E (µg/mL)	3.33 ± 1.56^b^	6.14 ± 1.50^b^	10.78 ± 4.18^a^	10.73 ± 4.52^a^	10.85 ± 3.57^a^

CNT=Control concentrate, LO=Control concentrate + lemuru fish oil 6%, LOE=LO + vitamin E 500 IU, LOS=LO + Se 0.50 ppm, LOES=LO + vitamin E 500 IU + Se 0.50 ppm. The same superscript in the same row indicates significant differences (p < 0.05) for treatments

Glutathione (GSH) levels significantly increased (p < 0.01) in LOE, LOS, and LOES groups compared to CNT and LO. Serum cortisol concentrations were significantly decreased (p < 0.01) across all supple-mented groups, with the greatest reduction in LOES (51.31 vs. 71.69 ng/mL in CNT). Serum Vitamin E levels were higher in LOE, LOS, and LOES than in CNT and LO (p < 0.01).

### Effects on growth performance

As shown in Table 8, none of the dietary treatm-ents significantly influenced final body weight, ADG, or FE (p > 0.05). Despite lower DMI in supplemented groups, growth performance remained comparable to that of CNT lambs, suggesting a compensatory effect of incre-ased dietary energy density.

**Table 8:** Effects of lemuru fish oil, selenium (Se), and Vitamin E on growth performance in male lambs.

Parameters	CNT	LO	LOE	LOS	LOES
Initial body weight (kg)	23.89 ± 2.99	22.60 ± 2.71	23.90 ± 2.34	23.94 ± 2.23	23.48 ± 2.50
Final body weight (kg)	25.44 ± 3.17	26.63 ± 2.74	24.67 ± 2.55	25.80 ± 3.04	25.72 ± 3.03
Average daily gain (g/h/d)	33.33 ± 22.05	53.00 ± 29.71	30.52 ± 30.44	52.19 ± 18.41	47.37 ± 24.54
Feed efficiency (%)	3.49 ± 2.31	6.63 ± 3.32	3.69 ± 3.58	6.36 ± 2.00	5.69 ± 2.81

CNT=Control concentrate, LO=Control concentrate + lemuru fish oil 6%, LOE=LO + vitamin E 500 IU, LOS=LO + Se 0.50 ppm, LOES=LO + vitamin E 500 IU + Se 0.50 ppm. The same superscript in the same row indicates significant differences (p < 0.05) for treatments

## DISCUSSION

### Nutrient intake and palatability

This study evaluated the effects of LO as a source of omega-3, combined with antioxidants such as Vitamin E and selenium, on lambs grown under high THI conditions. In this study, the hot climate during the dry season included severe heat stress in the morning, to extreme severe heat stress at noon and afternoon, as indicated by National Research Council (NRC) [15]. Under these conditions, animals were kept far from the thermoneutral zone, as classified by movement. The recommended ambient temperature for sheep is 20°C–30°C with a RH of approximately 60%. The average ambient temperature reached 42°C, accompanied by high levels of RH. This condition has implications for lambs that have experienced heat stress. Sufficient nutrient intake, immunological, physiological, and optimal growth could be challenging for lambs in this situ-ation [19, 20]. Therefore, we proposed a nutritional strategy to evaluate the effectiveness of LO alone or in combination with antioxidants in mitigating stress responses and improving lamb growth.

We hypothesized that LO alone or with antio-xidants would reduce heat increment and increase dietary energy density, thereby enhancing total DMI. However, the results only partially supported this hypo-thesis. Our results showed that feeding 6% LO in concentrate or combining LO with antioxidants decreased DMI. The reduced intake may be attributed to the fishy odor of LO, which could reduce palatability. The same result has been reported in another study on fish oil supplementation [21, 22]. However, another study reported that feed intake was not affected by 6% [23] or 3.9% [11] fish oil dietary supplementation.

Despite the lower intake, the total DMI (>760 g/day) and TDN intake (>500 g/day) in all treatment groups remained within NRC [15] recommendations for growing lambs under tropical conditions. However, the protein intake (71.58–90.42 g/day) was slightly below the NRC-recommended level (~97 g/day), indicating a potential area for adjustment in ration. These findings underscore the importance of careful formulation when incorporating fish oil into tropical feeding systems, particularly in balancing energy and protein while main-taining palatability.

### Feed affects physiological adaptations to heat

When exposed to high environmental temper-atures, sheep activate various physiological compe-nsatory mechanisms that help the body adapt to heat stress conditions, such as increased rectal temperature, respiration rate, and heart rate [24]. The heart rate of lambs receiving LO significantly decreased, with further reductions observed when Vitamin E and selenium were added. In this study, a decrease in rectal temperature was observed with the addition of LO with or without selenium and Vitamin E supplementation. Better physiological adaptation to heat stress responses in lambs supplemented with LO may be linked to dietary fat-induced lower heat increment. Dietary fat content helps reduce heat production [25]. Others reported that antioxidants do not affect the rectal temperature due to higher (approximately 10%) consumption in antioxidant treatment compared to CNT [26]. Our results indicate that the DMI was decreased by 11% in patients treated with LO and FO + antioxidants. The decrease in DMI and low heat increment may correlate with reduced heat production [26]. Selenium and Vitamin E can reduce the negative effect of heat stress on physiological responses in sheep [27] and goats [28]. To the best of our kno-wledge, this is the first study to demonstrate that combining antioxidants with fish oil is more effective in lowering heart rate under heat stress than fish oil alone.

### Hematological and immune responses

Blood hematological parameters are used as livestock health status markers. Our results show an increase in Hb and a tendency to increase the number of erythrocytes in livestock supplemented with LO. Supplementation with LO, whether alone or in combination with Vitamin E, selenium, or both, produced comparable effects on MCV and Hb conce-ntrations. An increase in red blood cells (RBC) with no increase in Hb was reported in cows supplemented with Se and Vitamin E [29]. RBCs were increased in lambs supplemented with selenium compared to Vitamin E [30]. In contrast, a study on lambs suppl-emented with selenium-enriched yeast reported no significant effect on Hb but observed a quadratic increase in RBC count with 0.6 mg selenium [31]. MCHC, a reliable red cell index, reflects hemolysis when elevated, while low values may indicate reticulocytosis or iron deficiency. Higher MCHC in our study correlated with higher Hb values. A higher MCHC value was also reported in lambs treated with organic selenium [32]. On the other hand, the MCV and MCHC showed an inverse trend. This may relate to the increase in Hb value in the treated group, but PCV was not affected. PCV in this study included a normal range compared to previous studies by Dias *et al*. [33] and Šimpraga *et al*. [34], and considered to be lower than the normal range, which started from 27% reported in another study in sheep [35]. PCV values vary depending on the intensity and duration of heat load experienced by the animal. Thus, sheep that suffer from prolonged periods of heat stress tend to decrease their PCV levels [36]. Changes in RBCs and indices (MCV and MCHC) are indications of adaptation from adverse environmental stress [37]. In other studies, by Indu *et al*. [38] and Sejian *et al*. [39], sheep that experienced heat stress had higher PVC, RBCs, and Hb. The different values of Hb and PCV are recorded between breeds in response to adaptive behavior to semi-arid conditions [36]. These findings underscore the need for additional studies on physiological adaptations to heat stress and nutrient interactions, particularly in Garut sheep.

In terms of leucocyte differentiation, lambs suppl-emented with LO and Vitamin E exhibited a higher perc-entage of lymphocytes. The ratio of neutrophils per lymphocyte was significantly decreased by only feeding LO. Adding antioxidants to LO does not result in a higher reduction in the N/L ratio. Heat stress can result in pathological atrophy of primary and secondary lymphoid tissues, leading to a decrease in lymphocytes and a degradation of their functional structure [40]. Higher lymphocyte count was reported and correlated with the beneficial effect of Vitamin E inclusion. High dietary Vitamin E supplementation enhanced *in vitro* lymphocyte proliferation in response to ConA and LPS mitogens, as shown by lymphoblastogenesis. Due to its antioxidant properties, Vitamin E may increase the function of lymphocytes by shielding them from lipid oxidation [41]. The lower ratio of neutrophils to lymphocytes when using LO or in combination with antioxidants indicates a reduced risk of negative effects of heat stress. An elevated N/L ratio is a recognized marker of physiological stress and impaired immune function [42]. Under heat stress, neutrophils tend to increase, while lymphocytes tend to decrease [43]. The decrease in N/L to approximately half of the CNT indicates the success of the treatment in enhancing the immune response to heat load in Garut lambs. A decrease in N/L ratio or better immunity was also reported in goats supplemented with L-glutamine, which has antioxidant properties [43].

### Antioxidant status

Heat stress negatively affects sheep’s oxidative status. Previous research has shown that hot enviro-nmental conditions promote oxidative stress and reduce antioxidant status by increasing the production of ROSs or impairing the antioxidant defense system, which significantly lowers blood concentrations of antioxidant capacity markers [27, 44-46]. Superoxide dismutase is the most important enzymatic antioxidant due to its ability to reduce ROS and radical chain reactions by repairing cells and slowing superoxide damage. The superoxide radicals are also transformed into more stable compounds [47]. The antioxidant enzyme SOD catalyzes the conversion of the extremely reactive O_2_• (superoxide anion) to O_2_, as well as to the less reactive H_2_O_2_ and peroxide, which can then be further broken down by GSH-peroxidase (GSH-Px) processes or CAT [48]. Vitamin E, a primary lipid peroxidation chain breaker, and selenium, a component of GSH-Px and thioredoxin reductase, play critical roles in regulating antioxidant activity by balancing the pro- and antioxidant status of the body [49]. Our hypothesis is that providing fish lemuru alone can result in better antioxidant status, and the effect becomes stronger when the LO is combined with Vitamin E and Se.

The combination of LO, selenium, and Vita-min E resulted in the greatest increase in SOD activity. Our results showed that adding Vitamin E with LO is not very effective in increasing SOD but remains higher than the CNT. However, as reported in cows, 0.3 ppm selenium and 1000 IU of Vitamin E were not effective in increasing SOD [29]. Our findings showed that supplementation with LO reduced SOD activity. Notably, the combination of LO with Vitamin E and selenium further reduced SOD by approximately 50% compared with the CNT group. Ambily *et al*. [29] reported that Vitamin E was the best to decrease SOD in cows compared to a combination of Vitamin E and selenium. This difference in results may be attributed to the higher Vitamin E dosage of 1000 IU/day compared to 500 IU/day.

This study demonstrated that supplementation with omega-3 fatty acids effectively reduced serum cortisol levels. Furthermore, adding Vitamin E and selenium to omega-3 produces superior effects. Cortisol is the primary indicator of hypothalamic-pituitary-adr-enal axis activation during stressful conditions, which can be increased by heat stress [50]. Heat stress activates the heat shock response, which promotes cytokine activity and impairs cellular immunity by increasing cortisol levels [51]. The effectiveness of omega-3 was reported on lambs fed 11% linseed oil, which could decrease cortisol levels after delivery stress [52]. Another study by Solanki and Devi [53] found that supplementing sheep with flaxseed, a form of omega-3, helped them cope with heat stress. A study by Xiao *et al*. [49] with dairy calves reported that Vitamin E decreases serum cortisol levels, indicating stress alleviation. Cortisol levels were significantly lower in sheep fed with selenium (0.3 mg) and Vitamin E (50 mg) per kg of diet for 4 weeks [54].

Although fish oil, Vitamin E, and selenium are known antioxidants, no significant effects on serum CAT activity were observed in this study. The addition of Vitamin E and selenium, and their combination with omega-3, promoted a higher concentration of GSH and Vitamin E in the serum. Higher GSH activity was expected in selenium-supplemented conditions bec-ause sele-nium is an essential constituent of GSH-Px. The primary constituent of GSH-Px, a member of the peroxidases group, is selenium [25]. As a donor of hydrogen atoms to cell membranes, Vitamin E (tocopherol) slows the peroxidation chain reaction in the lipid membrane [55]. Interestingly, this finding contrasts with previous repo-rts in which lambs fed solely on castor and cashew nut oils exhibited higher GSH-Px activity than those receiving oils in combination with antioxidants [56]. This discrepancy could be attributed to the different fatty acid contents of the LO used in this study.

### Interpretation of growth performance

Growth performance reflects both nutrient intake and the animal’s efficiency in using those nutrients. The results show that LO and its combination with antioxidants do not affect the growth performance para-meters. In contrast, another study indicated an increase in growth rate by dietary selenium and Vitamin E [50].

Notably, the reduced DMI observed in the treatm-ent groups may have limited the potential benefits of polyunsaturated fatty acid (PUFA) supplementation. However, despite the lower DMI in LO, LOE, LOS, and LOES, lambs maintained similar growth performance to the CNT group. This suggests that the dietary energy density from LO partially compensated for the lower intake.

From a practical perspective, these findings imply that lambs can maintain growth even under reduced feed intake during periods of heat stress when diets are developed with high-energy ingredients. While fish oil offers functional benefits, its palatability remains a limitation. Exploring alternative, palatable PUFA sources could enhance intake and promote better growth outcomes. In the context of climate change, where heat stress poses significant threats to livestock productivity, integrating heat-adaptive nutritional strategies may help mitigate economic losses and sustain small ruminant production systems in tropical regions.

## CONCLUSION

This study demonstrated that dietary supple-mentation with LO, alone or combined with antioxidants (Vitamin E and selenium), significantly influenced several physiological, hematological, antioxidant, and immune parameters in male Garut lambs exposed to high THI conditions. Notably, LO and antioxidant supplementation reduced rectal temperature and heart rate, improved antioxidant status as indicated by higher SOD and GSH levels, decreased serum cortisol, and enhanced hematological markers such as Hb and erythrocyte counts. Despite a reduction in DMI – likely due to the unpalatable odor of fish oil – the treated lambs maintained comparable growth performance to the CNT group, suggesting compensation through improved dietary energy density and physiological adaptation.

Practical implications of this study suggest that incorporating LO enriched with omega-3 fatty acids, especially in combination with Vitamin E and selenium, may serve as an effective nutritional strategy to mitig-ate heat stress and sustain health and performance in lambs raised in tropical environments. The results are particularly relevant given the growing concerns over climate change-induced thermal stress in small ruminant production systems.

The strength of this study lies in its comprehensive evaluation of nutrient intake, physiological responses, hematological parameters, immune markers, antio-xidant status, and growth performance under real-world heat stress conditions. Furthermore, the integration of both dietary fat and antioxidant interventions provides a novel and practical solution for improving therm-otolerance in sheep.

However, this study has some limitations. The observed reduction in feed intake due to fish oil pala-tability limits its scalability unless alternative flavor-masking strategies or more acceptable PUFA sources are explored. In addition, the study did not include histological or cellular-level assessments, which could have provided deeper insights into organ-specific stress responses and antioxidant activity.

Future research should focus on identifying more palatable sources of PUFA, optimizing the dosage and form of Vitamin E and selenium, and evaluating the long-term effects on reproductive performance, carcass traits, and meat quality. Additionally, molecular markers of heat stress and oxidative damage could be incorporated into future studies to better understand the underlying mechanisms.

LO, particularly when combined with Vitamin E and selenium, represents a promising nutritional intervention for alleviating heat stress and maintaining the physiological and metabolic health of lambs in tropical systems. This approach supports climate-resi-lient livestock production and offers a potential strategy for improving animal welfare and productivity under challenging environmental conditions.

## AUTHORS’ CONTRIBUTIONS

AN, AMD, SS, HH, FAP, and IA: Conceived, designed, and coordinated the study. AMD, FAP, DAA, and DD: Designed data collection tools. FIA, AN, ETS, and PIS: Supervised field sampling, data collection, laboratory work, and data entry. AMD, SS, AMD, IA, AN, and DAA: Statistical analysis and interpretation and drafted and revised the manuscript. All authors have read and approved the final version of the manuscript.
